# In vitro synergistic effect of hesperidin and doxorubicin downregulates epithelial-mesenchymal transition in highly metastatic breast cancer cells

**DOI:** 10.1186/s43046-023-00166-3

**Published:** 2023-03-27

**Authors:** Nur Dina Amalina, Irfani Aura Salsabila, Ummi Maryam Zulfin, Riris Istighfari Jenie, Edy Meiyanto

**Affiliations:** 1 Cancer Chemoprevention Research Center, Faculty of Pharmacy, Universitas Gadjah Mada, Sleman, Yogyakarta Indonesia; 2 Pharmacy Study Program, Chemistry Department, Faculty of Mathematics and Natural Sciences, Universitas Negeri Semarang, Semarang, Indonesia; 3 Department of Pharmaceutical Chemistry, Faculty of Pharmacy, Universitas Gadjah Mada, Sleman, Yogyakarta Indonesia

**Keywords:** Hesperidin; Doxorubicin; Cytotoxic synergism, Metastatic, 4T1 breast cancer cell

## Abstract

**Background:**

We previously reported that in highly metastatic breast cancer cells, doxorubicin (DOX) at non-toxic concentrations promoted cell migration and invasion. Hesperidin (30, 5, 9-dihydroxy-40-methoxy-7-orutinosyl flavanone) is a flavonoid glycoside isolated from citrus/lemon plant that possesses a cytotoxic effect in several cancer cells. In this study, we investigate whether DOX efficacy is enhanced by hesperidin (Hsd) and the molecular pathway involved in highly metastatic breast cancer, 4T1.

**Methods:**

Combined cytotoxicity of Hsd and DOX was evaluated with MTT assay and was analyzed using Chou-Talalay’s method. To better understand the underlying mechanism, several factors, including apoptosis and cell cycle arrest were analyzed by flow cytometry. In addition, antimigration activity was evaluated by scratch wound healing assay, MMP-9 expression by ELISA and gelatin zymography, and Rac-1 protein level using western blot. The data on survival rate and expression level of MMP-9 and Rac-1 were obtained from Gene Expression OMNIBUS (GEO).

**Results:**

Under MTT assay, Hsd showed a cytotoxic effect in a concentration-dependent manner with an IC50 value of 284 µM on 4T1 cells. Hsd synergistically enhanced the cytotoxic effect of DOX which seemed to correlate with an increase in apoptotic cell death, G2/M cell cycle arrest and blocked the migration of 4T1 cells. At 10 nM, doxorubicin induced lamellipodia formation, and increased the level of Rac-1 and metalloproteinase-9 (MMP-9) expression. Interestingly, combined treatment of DOX and Hsd dramatically downregulated the expression of MMP-9 and Rac-1. These results indicated that Hsd block the cell migration induced by DOX under in vitro studies.

**Conclusion:**

These findings strongly suggest that Hsd possesses a potential synergistic effect that can be developed to enhance the anticancer efficacy of DOX and reduce the risks of chemotherapy use in highly metastatic breast cancer.

## Background

Cancer cell metastasis is a complex process during which extracellular matrix degradation (ECM) plays a crucial role [1]. The MMP-2 and MMP-9 play a vital role in degradation of ECM and their function in the cancer metastasis cycle is complex [2, 3]. Moreover, Rac1, a Rho family GTPases, is also specifically involved in metastasis through induction of lamellipodia formation [4–6]. In recent years, the first line of metastatic cancer treatment still depends on chemotherapy such as doxorubicin (DOX) [7–9]. Unfortunately, DOX induces cancer cell resistance and more seriously also causes cell migration and invasion [10–12] through upregulation of myosin light chain 2 (MLC2) phosphorylation, the activity of RhoA [13] and TGF-ß1 signaling pathway activation to activated epithelial-mesenchymal transition (EMT) [14, 15]. Moreover, DOX significantly induced lamellipodia formation on highly metastatic and HER2-positive breast cancer cells [15]. Therefore, further studies need to be developed to explore the co-chemotherapeutic agent to inhibit the migration caused by doxorubicin.

Hesperidin (Hsd) is a citrus flavonoid glycoside that has been reported to show antiviral properties in silico [16] and cytotoxic effect in several cancer cells such as Hep-G2, HCT-116, MDA-MB-231, SK-N-MC [17, 18], T47D [19], Hela [20, 21], and MCF-7 [22, 23]. Hsd increased the cytotoxic effect of 5-Fluorouracil and DOX on human breast cancer cells or colon cancer cells, respectively [24, 25]. Based on these findings, Hsd appeared to increase sensitivity to DOX by downregulating the protein expression of pro-CASP3 and upregulating the level of active CASP3 [26–28]. Hesperidin inhibits the TGF-ß1/Smad signaling pathway [29]. Furthermore, in A549 cells, hesperidin shows a beneficial effect on the inhibition of EMT induced by TGF-ß1 [30]. In addition, the animal studies of Hsd did not indicate any toxic effects and were safe for therapeutical use [31]. However, the anti-metastatic effect and related mechanism in triple-negative breast cancer cells (TNBC) to inhibit the effect of doxorubicin inducing EMT is still unclear.

Thus, the current study looked into the effects of hesperidin as a co-chemotherapeutic agent in TNBC cells. Through several examinations, Hsd was evaluated for its effect on cytotoxicity, MMP-9 expression inhibition, cell migration inhibition, molecular marker, and bioinformatics study of TNBC metastasis. The findings of this study are expected to serve as a guideline for future research into hesperidin as a natural co-chemotherapy agent.

## Materials and methods

### Cell cultures

Strongly triple negative metastatic breast cancer cell line murine mammary carcinoma cell line from a BALB/cfC3H mouse (4T1 cells) was obtained from Dr. Masashi Kawaichi from Nara Institute of Science and Technology (NAIST), Japan. Cells were grown as a monolayer in Dulbecco’s modified Eagle medium (DMEM) high glucose (Gibco, USA) and added with 10% (v/v) fetal bovine serum (FBS) (Sigma, USA), 150 IU/ml penicillin-150 μg/ml streptomycin (Gibco, USA), 1.25 μg/ml amphotericin B (Gibco, USA) and maintained at 37 °C with 5% CO2 in 100% humidified atmosphere. For the experiment, 4T1 cells were used at a confluent of 80–90%.

### Cell viability assay

The proliferation of 4T1 cells was studied using MTT assay according to [32] with modification. 4T1 (2 × 10^3^ cells/well) were seeded in a 96-well microplate and allowed to settle overnight before the Hsd (Sigma-Aldrich, USA) (50–500 µM), DOX (Sigma-Aldrich, USA) (0.01–10 µM), and a combination thereof were applied. Cells were incubated for 24 h with Hsd, DOX, and its combinations treatment. Untreated cells were designed as negative control. After treatment, 100 µL of MTT (Biovision) (0.5 mg/mL in medium) was added into each well and further incubated for 4 h at 37 °C with 5% CO_2_. Afterward, the MTT formazan crystal was dissolved with a sodium dodecyl sulfate (SDS) stopper reagent containing 0.01 N HCl and incubated overnight in a dark condition. After solubilizing the purple formazan, the absorbance was measured using an ELISA plate reader (Corona SH-1000) at a wavelength of 595 nm. Each treatment was carried out in triplicate, and the cytotoxic activity was measured as the IC_50_, which is the concentration required to reduce the absorbance of cell population by 50% compared to the untreated cells. The cytotoxic combination assay to acquire the combination index (CI) value was determined using isobologram analysis. Combination index values determined using CompuSyn software based on the Chou-Talalay system show the effects of drug combinations [33, 34].

### Cell cycle analysis

Cell cycle analysis was performed by propidium iodide (PI) staining flow cytometry according to [35] with slight modification. 4T1 cells with a density of 2 × 10^5^ cells/well were cultured in a 6-well microplate in the presence of Hsd, DOX individually and its combination for 24 h. after treatment, all the media were eliminated, and the cells were trypsinized and centrifuged at 2000 rpm for 3 min. The collected cells were resuspended and fixed using ethanol for 30 min at 4 °C. Following that, the cells were washed with cold PBS and were centrifuged at 2000 rpm for 3 min. The cell pellet was resuspended in PI solution (50 μg/ml in PBS containing 1% Triton X-100 (Merck)) and DNase-free RNase A (20 μg/ml), and incubated for further 30 min at 37 °C in a dark condition. Finally, the cells were analyzed using flow cytometry (FACS Calibur, BD Biosciences, USA). After the cell debris was electronically gated out, the red fluorescence was calculated using the FL1 (log mode) setting. In addition, 20,000 events were measured with the Cell Quest Program (BD Biosciences) for subsequent study.

### Apoptosis assay

Apoptosis assay was performed using Annexin V-FITC/PI staining flow cytometry under Hsd, DOX, and a combination treatment. Briefly, the harvested cells were stained in an Annexin-V-FLUOS staining kit (Roche) composed of 100 ml binding buffer, 2 ml Annexin V and 2 ml PI for 10 min at room temperature in a dark place. The cells were measured using flow cytometry (FACS Calibur, BD Biosciences, USA) to measure the intensity of fluorescence using the using the parameter FL-1H to detect FITC. Further analysis to calculated the % apoptosis was done by using the Cell Quest Program (BD Bioscience) [36].

### Morphological lamellipodia formation

Lamellipodia formation assay was performed through microscopy study according to [15]. Cells were grown for 48 h and treated with Hsd, DOX, and the combination thereof. Every alternate day the medium containing Hsd, DOX, and the combination was changed. Representative image of the lamellipodia formation detected by inverted microscope (× 100 magnification).

### Scratch wound healing assay

The migration rate of the cells was assessed by the scratch wound healing assay under Hsd, DOX, and its combination treatment [37, 38]. Around 7.5 × 10^4^ cells/well of 4T1 cells were grown in a 24-well plate and incubated for 24 h at 37 °C and 5% CO_2_. After 80% confluent, the medium was discarded and changed with starvation medium containing 0.5% (v/v) FBS for 24 h. After that, the confluent cells were scraped horizontally with a cell scratcher after 24 h incubation. The debris was eliminated by PBS-washing, and the cells were treated with several concentrations of Hsd, DOX individually and in combination. Cell migration can then be observed microscopically (× 100 magnification) as the cells move from the intact zones into the scratched region. The migration rate was analyzed using ImageJ (Molecular Graphics Laboratory, The Scripps Research Institute) and the percentage of closure was measured and compared to the value obtained at 0 h. An increase in closure percentage suggested cell migration. Experiments were conducted in a triplicate, and the results were statistically reported and analyzed using SPSS with 95% percent reliance.

### Gelatin zymography assay

Gelatin zymography was performed to evaluate the activity of MMPs in the culture supernatants [39, 40]. The 4T1 cells density of 3 × 10^5^ cells/well were seeded in the 6-well microplate and exposed with Hsd, DOX, and a combination thereof for 24 h at 37 °C and 5% CO_2_. After the end of incubation, culture supernatants were collected and resolved on 8% SDS-PAGE under nonreducing conditions. The gel was supplemented with 0.1% gelatin (Sigma-Aldrich, USA). In each gel run, equivalent amounts of protein (150 μg) from culture supernatants were used. Gel were soaked for 30 min in 2% Triton-X 100 (Merck) solution after electrophoresis at room temperature before incubate in 100 mL of the incubation buffer contained 40 mM Tris HCl, pH 8, 10 mM CaCl_2_, 0.02% NaN_3_ for 24 h at 37 °C. Afterward, the gel was stained with Coomassie Brilliant Blue R-250 solution for 2 h and then de-stained (20% methanol, 10% acetic acid, and 70% water) until bands with a dark blue background appeared clear, indicating gelatinolytic activity of the pro MMP-9 and MMP-9. The band intensity was calculated by ImageJ (Molecular Graphics Laboratory, The Scripps Research Institute).

### ELISA assay

The pro MMP-9 ELISA assays were carried out using commercially available ELISA kits (Roche). In brief, as much as 2 × 10^3^ cells/well of 4T1 cells were treated with Hsd (35.5–142 μM). After that, the lysate of cells was transferred to a 96-well microplate precoated with pro MMP-9 specific antibodies. The lysate of cells was processed and measured according to the instruction of the manufacturer.

### Immunoblotting assay

Approximately 4 × 10^5^ cells/well of 4T1 cells were cultured in a 10-cm tissue culture dish, and after 24 h incubation, the cells were treated with Hsd, DOX, and a combination thereof for 24 h. The cultured cells were lysed in RIPA buffer (Tri-s-HCl pH 7.6, NP40, sodium deoxycholate, NaCl, SDS, phenylmethylsulphonyl fluoride (PMSF), NaF, and protease inhibitor cocktail. One hundred micrograms of protein lysates were separated by SDS-PAGE gel containing 10% acrylamide for β-actin analyses and 14% acrylamide for Rac-1 analyses. After electrophoresis, the gel was transferred onto a polyvinylidene fluoride (PVDF) membrane. The membrane was incubated overnight at 4 °C with the specified antibodies: Rac-1 (ab33186, Abcam) and β-actin (sc-47778, Santa Cruz), followed by incubation with anti-mouse secondary antibody (sc-516102, Santa Cruz) for 1 h at the room temperature. Protein was visualized using Luminograph (Atto) or enhanced immunoreactive band by electrochemiluminescence (ECL) reagent (#1,705,062, Biorad or ECL Prime, Amersham Bioscience). The quantify of western blot expression was calculated by analysing band intensity using ImageJ software, and The relative protein content were displayed in proportion to the amount of β-actin protein [37].

### Microarray analysis

Bioinformatics study of Rac-1 and MMP-9 gene expression. Profiling data were collected from the NCBI Gene Expression Omnibus (GEO, http:/www.ncbi.nlm.gov/geo/) for Rac-1 and MMP-9 gene expression. With regard to the differential expression study of each gene in TNBC and Non-TNBC cancer cells, GEO datasets accession numbers GDS5437/1451086_s_at (Race-1) and GDS5437/1416298_at (MMP-9) were obtained and statically analyzed under R version 3.2.2.

### Molecular docking simulation

To verify the binding interaction between Hsd toward MMP-9 and IKKB, we performed a molecular docking analysis using MOE 2010.10 (licensed to Faculty of Pharmacy, UGM). This assay was conducted to simulate molecular binding, calculate RMSD, and visualize protein–ligand interaction. The PDB ID of MMP-9 and IKKB were 2OW1 and 4KIK, respectively. The triangle matcher and London ΔG were used for placement and scoring. The force field method was used to refine the docking results from ten retain settings. Then using a chemical builder in MOE, we visualize the structure of Hesperidin. The structure is then prepared for structural energy minimization and generated for conformational structure in MOE. The molecular docking study was conducted on each protein's native ligand binding site. The molecular docking result explained the affinity represented by the docking score as well as the binding visualization of each compound to the target proteins.

### Statistical analysis

All values depicted represent the mean ± SD of three independent experiments. For statistical comparisons, the one-way variance analysis (ANOVA) was performed followed by the least significant difference (LSD) post-hoc test using SPSS 21 software and GraphPad Prism 9.2.0.

## Results

### Inhibition of metastatic breast cancer 4T1 cells growth by Hsd dan DOX individually and in combination thereof

**Fig. 1:**
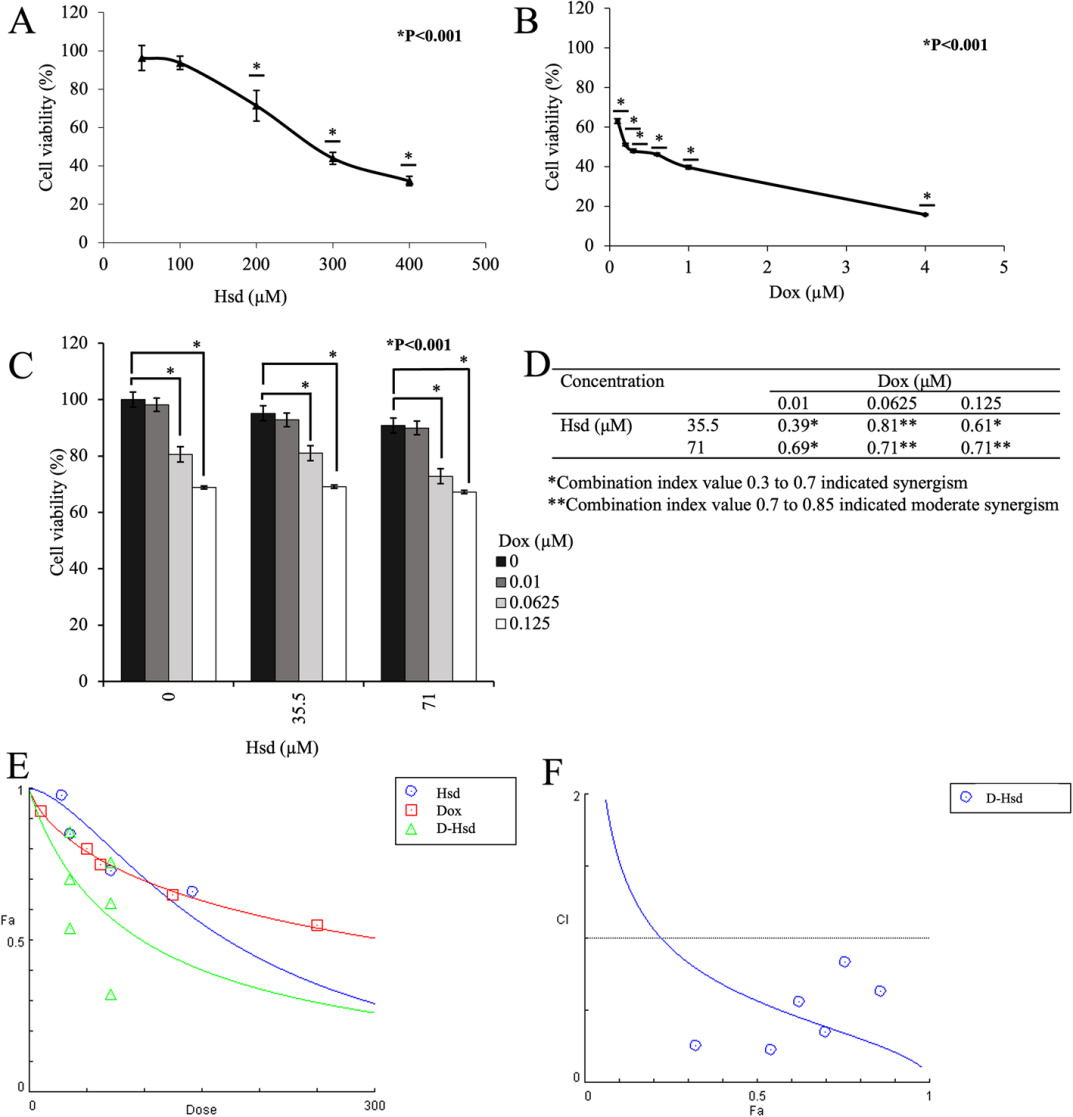
Synergism cytotoxicity of Hsd and DOX on 4T1 breast cancer cells. 4T1 cells were treated individually or in combination with Hsd plus DOX at various concentrations for 24 h, and the viability of cells was evaluated using MTT assay. Percentage of cell viability under **A** Hsd and **B** DOX, **C** combination of Hsd-DOX treatment for 24 h. Cell viability profiles were expressed as mean ± SD from three independent experiments. **D** The combination index (CI) value of various combination ratios. CI value below and above CI = 1 will indicate synergism and antagonism, respectively. **E** Dose–effect curves of Hsd, DOX, and its combination. Dose–effect curves were generated from the CompuSyn calculation, and the values are the mean of three experiments. **F** Combination index plot among 6 combinations, all of the data were on the synergistic side (CI < 1). *p < 0.001 (post-hoc LSD test between each group) was considered statistically significant

The cytotoxic activity of Hsd and DOX on the cell viability of 4T1 metastatic breast cancer cells was evaluated using an MTT assay. Various concentrations of Hsd and DOX were used for 24 h treatment of 4T1 cells. After exposure to Hsd and DOX, the proliferation of 4T1 cells was inhibited in a concentration-dependent manner with an IC_50_ value of 284 µM (Fig. 1A) and 0.5 µM (Fig. 1B), respectively. As our previous results indicated, the IC_50_ value of Hsd exhibited mild cytotoxicity, in contrast with the IC_50_ value of DOX, indicating exhibited a strong cytotoxic effect. However, our previous result reported that DOX-induced lamellipodia formation through activation of TGF-ß signaling to trigger Epithelial-mesenchymal transition (EMT) formation, induced invasion, and enhanced generation of cells with stem cell phenotype. This phenomenon leads to a decrease in the efficacy of DOX. Then we conducted a combination treatment for further exploration with the aim of increasing the efficacy of therapy and reducing side effects. As shown in Fig. 1C, compared to Hsd alone, the combination of Hsd and DOX induced significantly higher cytotoxicity in 4T1 cells. Furthermore, a study of Chou-Talalay was used to assess whether Hsd synergized with DOX to induce enhanced cytotoxicity. The results of the combination index (CI) for various combinations of Hsd and DOX are summarized in Fig. 1D–F. All combinations demonstrated synergism with a CI value < 1. These findings indicated a synergistic effect of low concentration Hsd and DOX on inhibiting the viability of 4T1 cells. Our primary objective in this study was to detect whether the low dose of DOX is still able to function effectively in human breast cancer cells due to increasing cell sensitivity by Hsd. For our subsequent experiments, we selected 10 nM of DOX and one-half or one-fourth IC_50_ of Hsd, both of which showed low toxicity when used individually and dramatically exhibited synergistic effect when used in combination.

### Hsd and DOX individually and in combination induced apoptosis in 4T1 cells

**Fig. 2:**
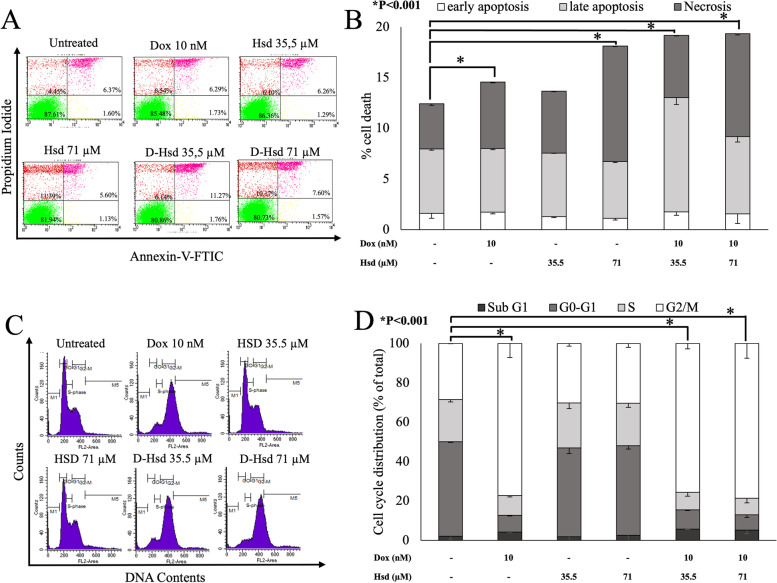
Induction of apoptosis and cell cycle distribution by Hsd, DOX, and combination treatment on 4T1 breast cancer cells. Cells were exposed with Hsd and DOX individually or in combination for 24 h. % cell death (**A**, **B**) and % cell distribution in each phase (**C**, **D**) were measured by flow cytometry after staining with Annexin V-FITC/PI and PI, respectively. The bar represents the mean ± SD value from the triplicate independent experiment. *p < 0.001 (post-hoc LSD test between each group) was considered statistically significant

To observe the underlying mechanism of cytotoxic activity induced by Hsd and DOX treatment was related to apoptosis induction was assessed using flow cytometry. The % cell death of 4T1 cells was observed by treating with 10 nM DOX, one-half IC_50_ Hsd, one-fourth IC_50_ Hsd and its combination for 24 h using flow cytometry Annexin V-FITC/PI staining. Four separate cell populations are obtained according to this method. Annexin V negative-PI negative indicated live cells, Annexin V positive-PI negative indicated early apoptosis, Annexin V positive-PI positive indicated late apoptosis, and also Annexin V negative-PI negative indicated necrotic cells (Fig. 2A). No obvious apoptosis effects were observed in one-fourth IC_50_ Hsd compared with untreated. However, treatment with 10 nM DOX and one-half IC_50_ Hsd individually induced apoptosis up to 14.67% and 18.12%, respectively. In addition, the combination of 10 nM DOX-one-fourth IC_50_ Hsd (35.5 µM) and 10 nM DOX-one-half IC_50_ Hsd (71 µM) increased apoptosis by 19.17% and 19.34%, respectively (Fig. 2B). Based on apoptosis induction, a further study needed to be conducted to explore the effect of Hsd in combination with DOX on cell cycle arrest.

### Hsd and DOX individually and in combination induced cell cycle arrest in 4T1 cells

Besides induction of cell death, improvement of the combination effect could also occur by modulation of the cell cycle. Flow cytometry was used to determine cell cycle distribution after treatment. 4T1 cells were treated with 10 nM DOX, one-half IC_50_ Hsd, one-fourth IC_50_ Hsd and its combination for 24 h. The results in Fig. 2C, D showed no effect on cell cycle distribution were treated with Hsd in two concentrations. In comparison, DOX alone or in combination with Hsd resulted in an increase of cell cycle arrest at the G2/M phase up to 50.12% compared to untreated. Cell accumulation also increased during combination treatment at G0/G1 phase.

This phenomenon is probably caused by the differences in target content due to the fragmentation of DNA. Consequently, the combination of Hsd-DOX increased 4T1 cell death through induction of cell cycle arrest and apoptosis. Further study was required to investigate Hsd activity in inhibition of DOX-induced migration.

### Hsd inhibited lamellipodia formation and migration of cells

**Fig. 3:**
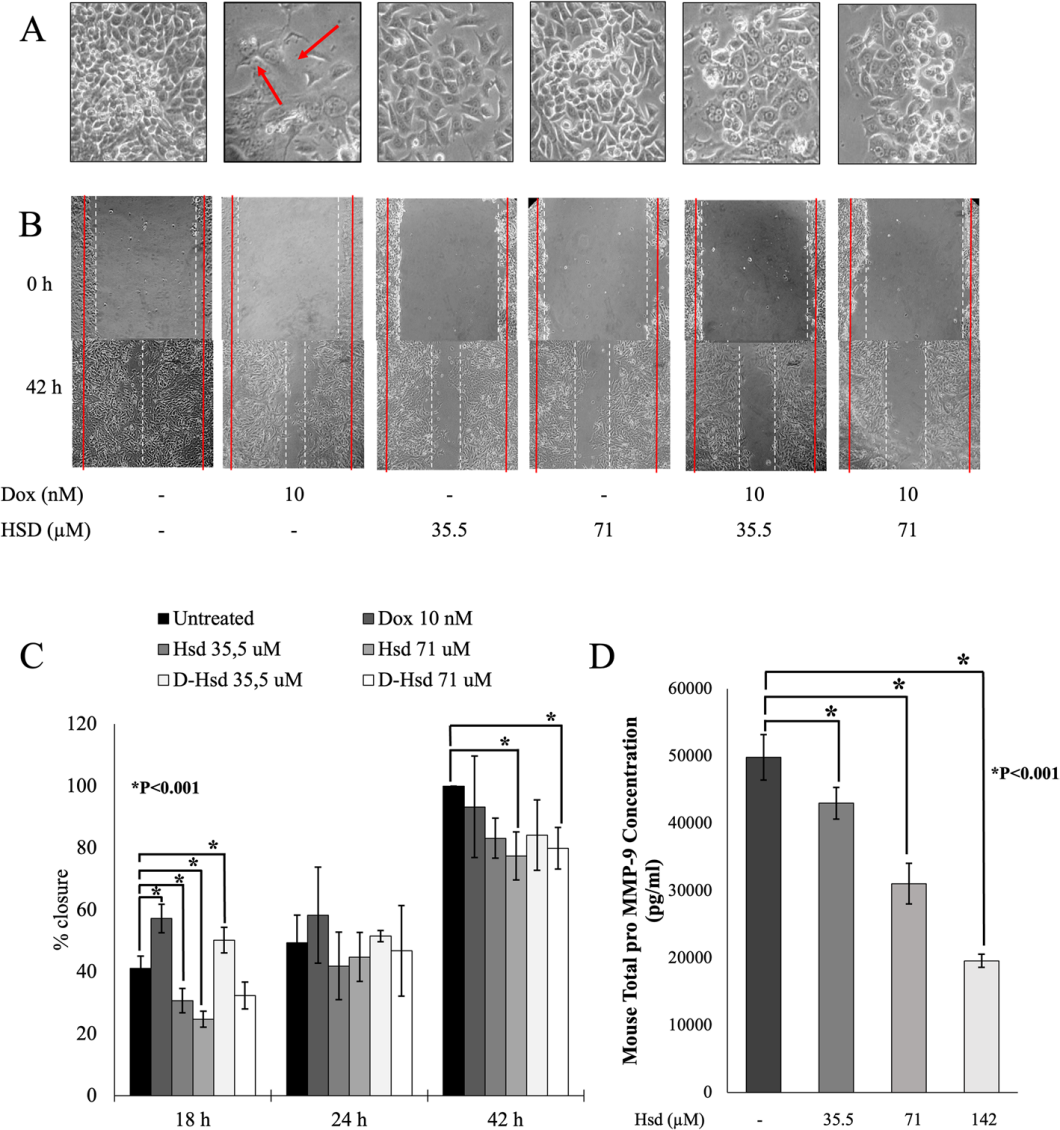
Effect of Hsd, DOX, and combination treatment on the migration ability of 4T1 breast cancer cells. **A** After treatment, the formation of lamellipodia was observed under inverted microscopy (× 100 magnification). The red arrows that there are morphological differences. **B** Cell migration was assessed via scratch wound healing assay. **C** The migration abilities were quantified by counting the area of closure using ImageJ, and the percentage of the treated group compared to the untreated group is presented as the mean ± SD of the triplicate independent experiment. **D** The expression level of pro MMP-9 protein was measured by ELISA. The quantitative data were shown as the mean ± SD of the triplicate independent experiment. *p < 0.001 (post-hoc LSD test between each group) was considered statistically significant

Since cell migration is a key process in metastasis and tumor invasion, the effect of Hsd on the migratory potential of highly metastatic breast cancer 4T1 cells was evaluated using lamellipodia observation and in vitro scratched wound healing assay. Lamellipodia observation is used to check the ability of cells to migrate under an inverted microscope. The findings showed that DOX 10 nM induced strong reorganization of the actin cytoskeleton in prominent lamellipodia formation (Fig. 3A). Even though Hsd at concentrations one-half and one-fourth of IC_50_ did not alter cell morphology, the combination treatment significantly reduced the elongation of lamellipodia induced by DOX. This phenomenon suggested that Hsd showed potent inhibition of DOX-induced lamellipodia formation.

Furthermore, we observed the ability of 4T1 cell migration to support this finding by analyzing scratch wound healing assay. The 4T1 cells were classified as a model of the TNBC and for their metastatic potential. Untreated cells in which the wound width narrowed at 24 h and closed completely throughout 42 h (Fig. 3B). Treatment with Hsd found to be significantly inhibited the migration of 4T1 cells in a concentration-dependent manner. When the cells were treated with DOX (10 nM), 58% wound closure was observed at 24 h, and at 42 h wound was almost completely closed. Interestingly, at a combination of 10 nM DOX-one-fourth IC_50_ Hsd and 10 nM DOX-one-half IC_50_ Hsd, 4T1 cells showed 84% and 80% for 42 h of inhibited wound closure when compared to untreated (Fig. 3C). The results indicate the antimigratory potential of Hsd against 4T1 cells due to DOX-induced migration.

Hence, the inhibitory effect of Hsd needs to be studied further for the pro-MMP 9 activity using ELISA. Under Hsd treatment, the concentration of Pro MMP 9 in the supernatant medium was found to be reduced in a concentration-dependent manner. Hsd at concentration 35.5, 71, and 142 μM inhibited 13.67%, 37.68%, and 60.68% pro MMP 9 expressions, respectively (Fig. 3D). Furthermore, to explain the mechanism underlying the antimigratory effect of Hsd, we observed the MMP 9 activity and Rac1 protein expression.

### Hsd inhibited MMP 9 and Rac1 expression

**Fig. 4:**
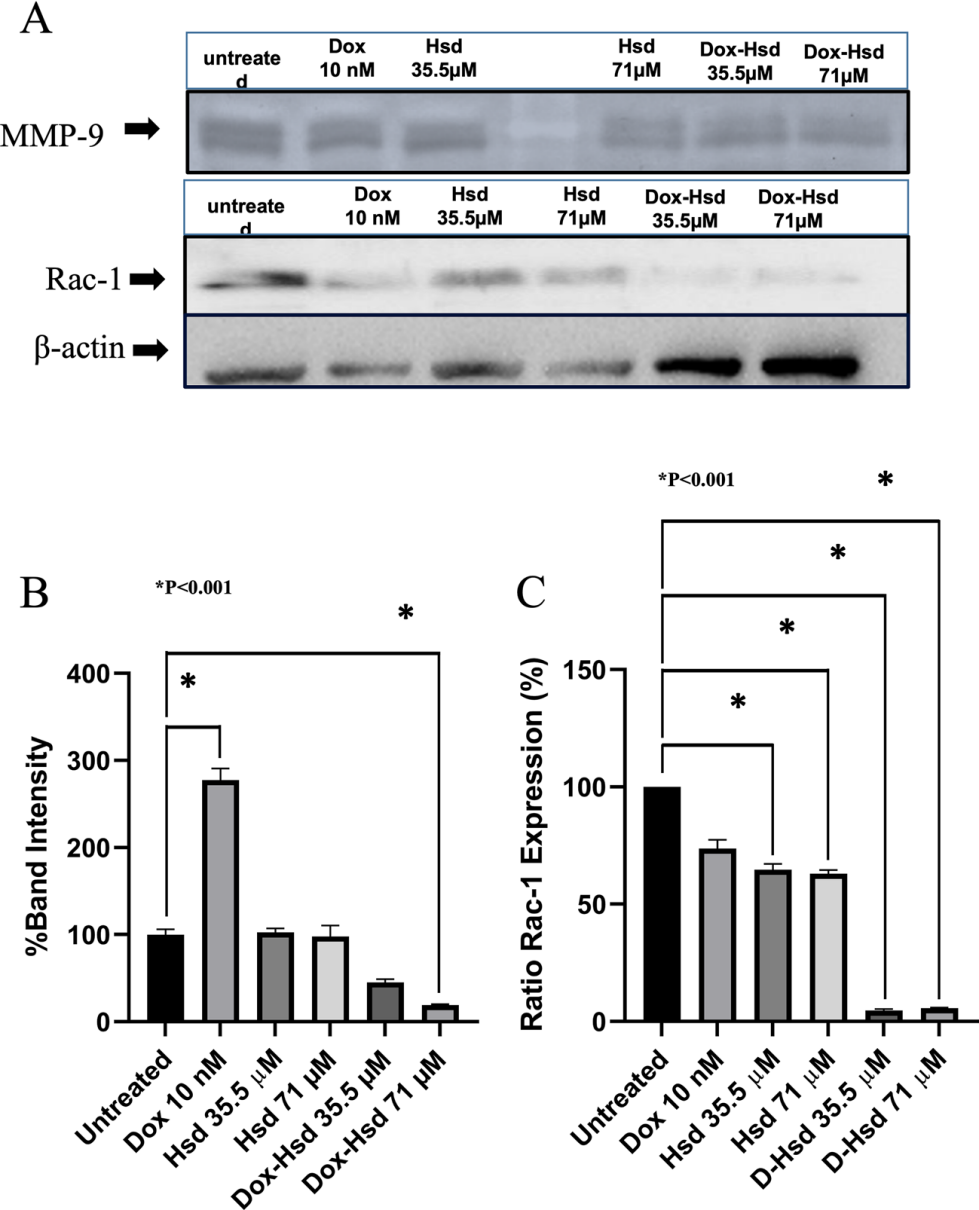
The activity of MMP-9 and the expression of Rac-1 after Hsd, DOX, and combination treatments on 4T1 breast cancer cells. The 4T1 cells were incubated with test samples for 24 h for further isolation and assays. **A** The MMP activity of MMP 9 and pro MMP 9 were analyzed using gelatin zymography. **B** The quantification of MMP 9 expression levels under gelatine zymography. **C** Rac-1 expression level of cell migration-related proteins was measured by western blotting. The quantification of band intensity was calculated with ImageJ software and presented as the graph ratio compared with untreated. The data were shown as the mean ± SD of a triplicate independent experiment. *p < 0.001 (post-hoc LSD test between each group) was considered statistically significant

MMP 9 activity inhibition is a key step towards elucidating the inhibition of the migration process, while Rac1 tightly regulated the formation of lamellipodia. Gelatin zymography was conducted to evaluate Hsd, DOX, and the combination thereof on the inhibition of MMP 9 activity. There is no DOX effect on MMP 9 expression reductions. On the other hand, Hsd significantly decreased the expression of MMP 9 in a concentration-dependent manner. More importantly, Hsd also effectively blocked the expression of MMP 9 in combination treatment compared with DOX-treated and untreated cells (Fig. 4A, B). Single Hsd treatment and its combination with DOX thus suppressed the expression of MMP 9.

Western blotting had calculated the influence of Hsd on the expression of Rac1. DOX and Hsd individually reduced the expression of Rac1 compared with untreated cells. In other words, a combination of DOX-Hsd significantly decreased Rac1 expression (Fig. 4C). More importantly, in decreasing Rac1 expression, a single treatment of Hsd was more effective than the DOX treatment, and a combination of Hsd 35.5 µM and DOX 10 nM resulted in the lowest expression of Rac1. Overall, Hsd reduced Rac1 expression, which is related to the anti-metastatic activity on DOX-induced metastasis breast cancer cells.

### Protein targets of Hsd on metastatic breast cancer and its molecular interaction with Hsd

**Fig. 5:**
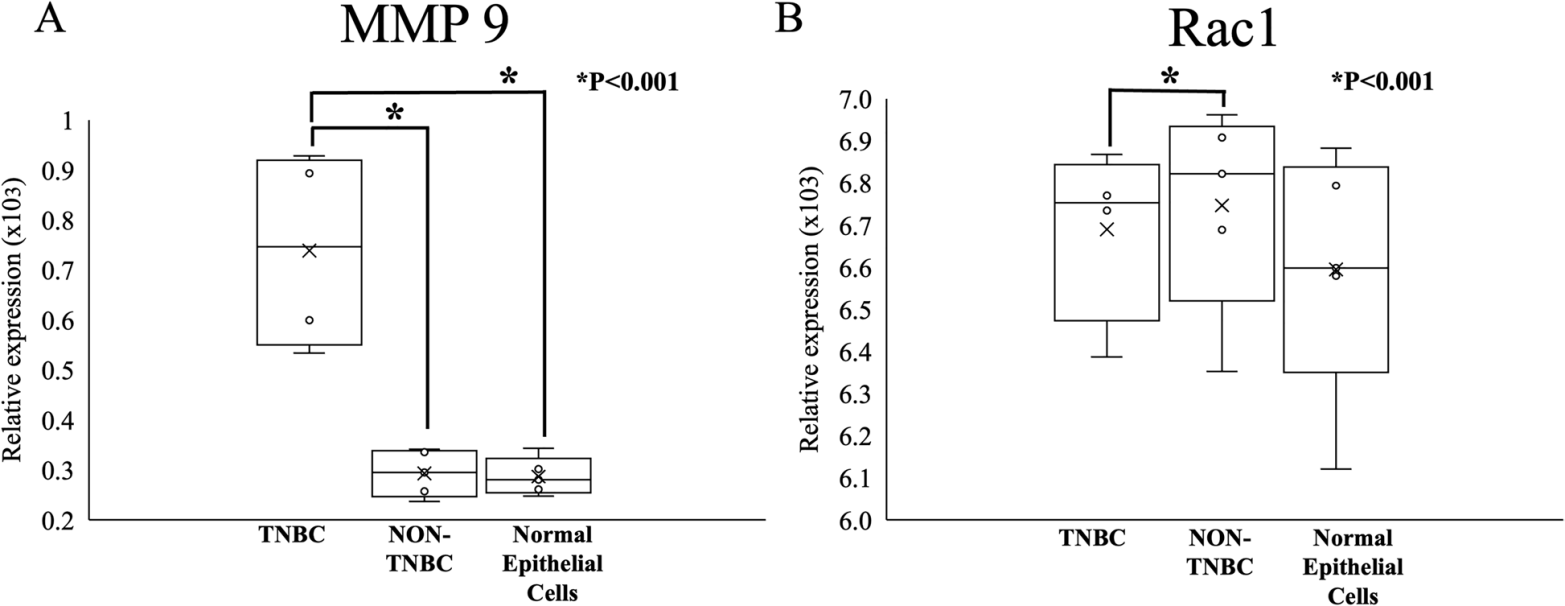
Analysis of Hsd interactors in TNBC by Microarray data analysis. **A** MMP 9 expression. **B** Rac1 expression. Gene expression data were collected from the GEO datasets. *p < 0.05 (the Kruskall-Wallis test) was considered statistically significant

To confirm the inhibition phenomenon, we also analyzed the expression level of Hsd interactors (MMP 9 and Rac1) in a gene expression omnibus (GEO) dataset of cases of TNBC, non-TNBC and normal cells. MMP 9 overexpressed in TNBC (*P* < 0.001), while the expression of Rac1 revealed no significant difference between TNBC and normal cells (Fig. 5A, B).

**Fig. 6:**
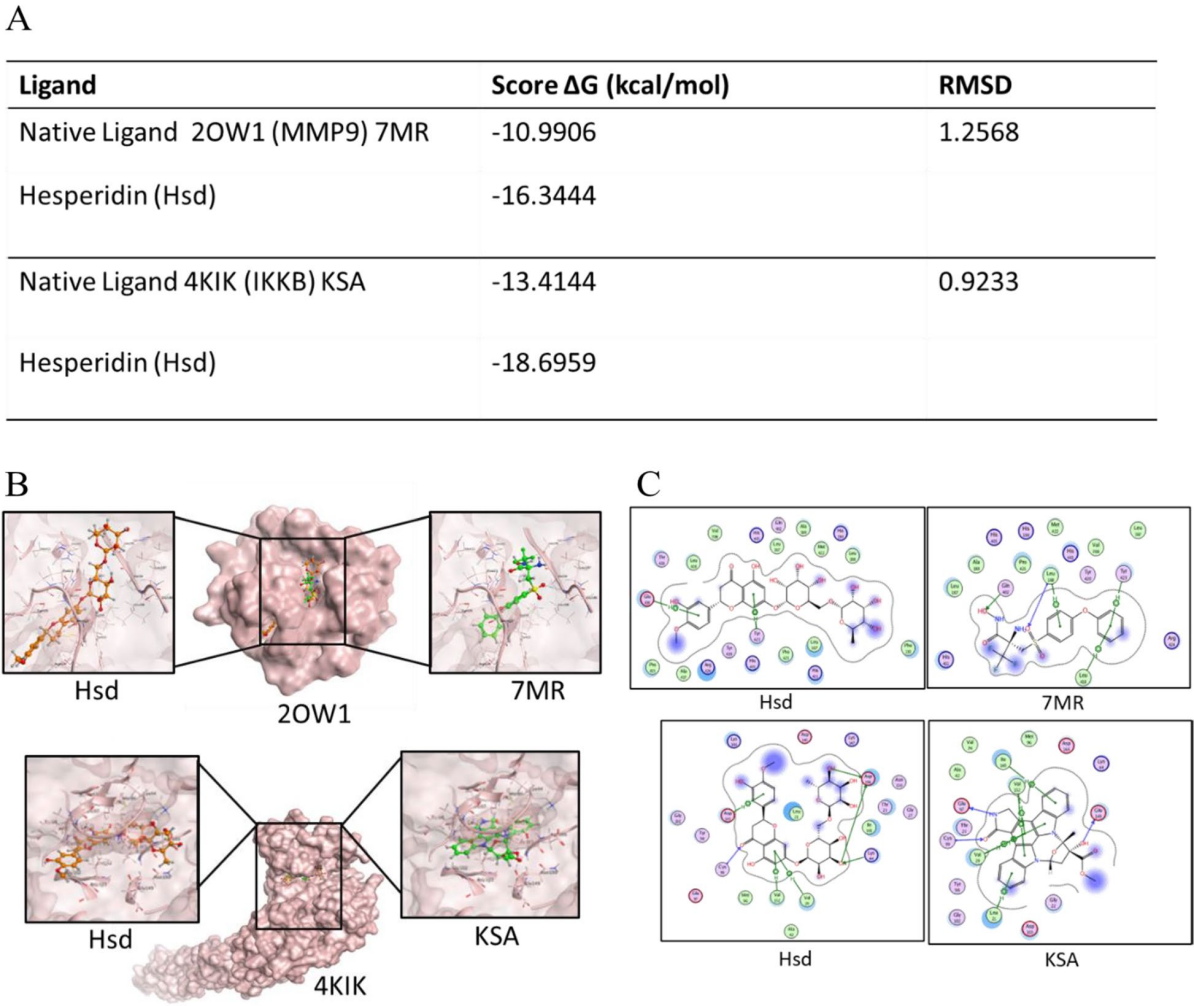
The molecular docking simulation of Hsd towards MMP 9. **A** Docking score of Hsd, native ligand 7MR, and native ligand KSA. **B** 3D visualization of Hsd and each native ligands of 2ow1 (crystal protein of MMP 9) and 4KIK (crystal protein of IKKB). **C** 2D visualization of Hsd interaction towards MMP 9 and IKKB

Further, the docking simulation of Hsd towards MMP 9 protein was evaluated using MOE Software. The protocol was considered to be valid based on its RMSD value (1.2568). Hsd with a docking score of − 16.3444 performed a strong interaction towards 2OW1 protein, a crystal protein of MMP 9, compared to its native ligand with a docking score of − 10.9906. Furthermore, Hsd showed a strong interaction as well towards IKKB protein, an upstream protein of MMP 9 (Fig. 6A). The interaction between Hsd and specific amino acid residues at MMP 9 pocket contributes to the strong interaction, which leads to the inhibition effect of Hsd towards MMP 9 as well as IKKB (Fig. 6B, C).

## Discussion

Despite the fact that DOX is an effective method of extending survival and lowering mortality, several studies reported that DOX usage leads to metastatis [14, 41]. It is important to explore the compound that may inhibit DOX-driven metastatic and increase the cytotoxicity of DOX. Therefore, in this study, we focus on evaluating the anti-metastatic activity of Hsd to inhibit DOX-induced metastatic on triple negative breast cancer model, 4T1 cells. We found that Hsd, a flavanone glycoside found in citrus fruits, had a cytotoxic effect in a concentration-dependent manner, while in combination with DOX, Hsd significantly enhanced the cytotoxic effect of DOX with the synergism effect of combination treatment [37]. This finding suggested that Hsd may increase cell sensitivity to DOX to promote optimum cytotoxic activity while inhibiting metastatic effect [42, 43]. The increased cytotoxic activity of Hsd in combination with DOX was potentially caused by several factors as Hsd and DOX were simultaneously internalized in the cells, this allowed both compounds to supplement the antitumor effects of the other compounds. In addition, these results provide a general description that Hsd is promising to be developed as a co-chemotherapeutic agent for inhibiting DOX-driven metastatic.

Furthermore, we explored the underlying mechanism of physiological changes due to the Hsd and its combination treatment. We found that in combination treatment, Hsd-DOX significantly promoted an even higher evidence of apoptosis than the Hsd/DOX single treatment. The result of the apoptosis assay showed that Hsd-DOX was giving a synergistic effect. In addition, compared to DOX, a single treatment of Hsd yielded a stronger apoptosis effect. This suggests that the apoptosis effect in combination is predominantly due to the presence of Hsd. A previous study confirmed that Hsd also strongly induces apoptosis on A549 lung and Hela cells [21, 44, 45]. Apoptosis induction is one of the therapeutic strategies for cancer cure that lead to concentrating on the programmed cell death pathway, which is controlled by genes that keep the internal environment stable. The apoptosis rate is an important metric for assessing the therapeutic efficacy of anticancer agents. On the other hand, the combination treatment of Hsd-DOX caused cell to remain in the accumulation in the G0/G1 and G2/M phases. These results indicated that the combination treatment induced cell arrest at G0/G1 and G2/M phases, continued by significant apoptotic cell death in 4T1 cells. Similarly, Hsd and DOX have also been known to cause G0/G1 and G2/M arrest in human lung and breast cancer cells, respectively, by increasing the level of p21 and decreasing the level of cyclin B1 and cyclin D protein in a p53-dependent manner [46, 47]. Considering that, an alteration in cell apoptosis and cell cycle distribution occurred following combination therapy.

During the progression of cancer, certain tumor cells become motile and attack the host tissue, resulting in metastatic disease. Metastasis may arise due to the DOX administration by an increased level of TGF-β and circulating cancer cells on metastatic lung breast cancer cells [48, 49]. In this process, TGF-β activation may drive lamellipodia formation leading to inhibition of wound closure. The formation of lamellipodia requires Rac1 protein and MMP-9 activities, which play a role in tumor invasion [50]. MMP-9 activity is linked to cancer occurrence and progression, DOX promotes MMP-9 activity in human breast cancer cells by regulating the integrin pathway and proteolytic cleavage [51, 52]. In addition, Rac1 expression increases myosin phosphorylation, which cross-links actin filaments and generates contractile force, promoting cell body movement during cell invasion [53, 54]. Active Rac-1 could promote endothelial cell migration by inducing MMP-9 expression.

One of the interesting aspects of Hsd is that it inhibits migration and lamellipodia formation through downregulation of MMP 9 and Rac1 protein [2, 50]. All of the proteins mentioned above are part of signalling networks that have metastatic effects, and Hsd was discovered to interfere with these signalling networks in order to reduce DOX-induced migration. More specifically, in this study, DOX-induced metastatic potential was confirmed by inhibited wound closure rate and induction of lamellipodia formation by upregulation of MMP 9 and Rac1 protein expression after DOX treatment alone without affecting cell viability. Some previous studies revealed that the DOX administration could induce the migration of highly metastatic breast cancer 4T1 cells and MCF7/HER2 + breast cancer cells [15, 55] by activating the TGF-β1 pathway leading to EMT [14]. EMT is the process by which epithelial cells decide to become mesenchymal-like cells, and it is a critical step in the formation of lamellipodia, which leads to cancer metastasis. Afterward, co-treatment with Hsd significantly reversed the inhibition in wound closure and decreased lamellipodia formation by downregulation of MMP-9 and Rac-1 protein expression. The inhibition of cell migration by Hsd might be correlated to a decrease in the level of TGF-β protein leads to NF-κB pathway inactivation, which is an important contributor to block metastasis [56]. As a result, the mechanism by which Hsd inhibits migration in 4T1 cells is most likely due to TGF- and NF-B signalling inhibition; however, this hypothesis requires further investigation.

Metastatic breast cancer cells characterize more by surviving against oxidative conditions and exhibit resistance to chemotherapeutic agents leading to a high incidence of cancer relapse. Several chemotherapeutic agents aggravate this condition through activation of EMT, leading to cancer cell migration. Additional findings of the synergistic effect indicated that a combination of Hsd and DOX is able to elevate the cell death number through apoptosis induction and cell cycle arrest modulation in highly metastatic breast cancer cells. Increased levels of MMP-9 and Rac-1 protein expression under DOX administration caused the lamellipodia formation, causing EMT leading to progressive metastatic. However, the effect of Hsd in lowering cell migration indicates its potential to reduce the side effect of DOX-induced metastatic. Interestingly, in this study, combination therapy of Hsd-DOX significantly inhibits the migratory capacity of DOX through lamellipodia, MMP-9, and Rac-1 protein regulation. These results showed that the anti-metastatic effect of Hsd may be dependent on lamellipodia formation, MMP-9, and Rac-1 protein expression.

Taken together, Hsd inhibited the metastatic ability of DOX-induced highly metastatic breast cancer cells in vitro by lowering MMP-9 and Rac-1 expression, which is closely integrated with cytotoxic activity, apoptosis, and cell cycle regulation. Collectively, these finding denotes the potential of Hsd as a natural co-chemotherapeutic agent to reduce the side effect of DOX-induced migration and prevent cancer recurrence.

## Conclusions

To summarize, our research demonstrated that Hsd, in combination with DOX, significantly inhibits 4T1 cell growth, induces apoptosis and G2/M cell cycle arrest, and inhibits cell migration by suppressing the expression of MMP-9 and Rac-1 proteins. In addition, the combination of Hsd and DOX demonstrated a synergistic inhibitory effect on the 4T1 cell growth. Our interesting findings show that Hsd is potential to be developed as a co-chemotherapeutical agent that might resolve cancer metastasis.

## Data Availability

The authors confirm that the data supporting the findings of this study are available within the article.

## Abbreviations

DOX: Doxorubicin
Hsd: Hesperidin
GEO: Gene Expression Omnibus
MMP-9: Matrix mettaliproteinase-9
ECM: Extracellular Matrix
MLC2: Myosin Light Chain 2
EMT: Epithelial-mesenchymal Transition
TNBC: Triple Negative Breast Cancer Cells
PI: Propidium Iodide
PMSF: Phenylmethylsulphonyl fluoride
PVDF: Polyvinylidene fluoride
ECL: Electrochemiluminescence
TGF-β: Transforming growth factor beta
NF-κB: Nuclear factor kappa-light-chain-enhancer of activated B cells
